# Facial Expression Related vMMN: Disentangling Emotional from Neutral Change Detection

**DOI:** 10.3389/fnhum.2017.00018

**Published:** 2017-01-30

**Authors:** Klara Kovarski, Marianne Latinus, Judith Charpentier, Helen Cléry, Sylvie Roux, Emmanuelle Houy-Durand, Agathe Saby, Frédérique Bonnet-Brilhault, Magali Batty, Marie Gomot

**Affiliations:** ^1^UMR 930 INSERM, Université François-Rabelais de ToursTours, France; ^2^Centre Universitaire de Pédopsychiatrie, CHRU de ToursTours, France

**Keywords:** visual mismatch negativity, equiprobable, emotion, anger, face, EEG

## Abstract

Detection of changes in facial emotional expressions is crucial to communicate and to rapidly and automatically process possible threats in the environment. Recent studies suggest that expression-related visual mismatch negativity (vMMN) reflects automatic processing of emotional changes. In the present study we used a controlled paradigm to investigate the specificity of emotional change-detection. In order to disentangle specific responses to emotional deviants from that of neutral deviants, we presented neutral expression as standard stimulus (*p* = 0.80) and both angry and neutral expressions as deviants (*p* = 0.10, each). In addition to an oddball sequence, an equiprobable sequence was presented, to control for refractoriness and low-level differences. Our results showed that in an early time window (100–200 ms), the controlled vMMN was greater than the oddball vMMN only for the angry deviant, suggesting the importance of controlling for refractoriness and stimulus physical features in emotion related studies. Within the controlled vMMN, angry and neutral deviants both elicited early and late peaks occurring at 140 and 310 ms, respectively, but only the emotional vMMN presented sustained amplitude after each peak. By directly comparing responses to emotional and neutral deviants, our study provides evidence of specific activity reflecting the automatic detection of emotional change. This differs from broader “visual” change processing, and suggests the involvement of two partially-distinct pre-attentional systems in the detection of changes in facial expressions.

## Introduction

The visual system has limited capacity to process information from a constantly changing environment. Automatic detection of relevant stimuli is thus a fundamental requisite to (re)orient attention and, for instance avoid danger (Corbetta and Shulman, 2002). To this aim, attentional systems rapidly and automatically process “signal” stimuli, which can be either novel/changing events, or emotional stimuli. Yet, the interplay between the automatic attention systems dedicated to either changing or emotional stimuli, remains poorly understood (Brosch et al., 2011).

Facial expressions provide socially relevant cues, which can be used to study emotion-related attentional mechanisms. Faces convey rapid information about others' mental states and intentions, playing a central role in social reward and decision making (Bechara, 2004). Emotional expressions are crucial signals that communicate possible threats in the environment (Anderson et al., 2003). For these reasons, an emotional facilitation effect has often been reported, showing that even when attention is engaged in a concurrent task, emotional information is prioritized and automatically processed (Ikeda et al., 2013; Carretié, 2014; Hinojosa et al., 2015). Experimental studies have investigated face processing by analyzing behavioral and neural responses to emotional faces. Several neuroimaging studies have shown that sensory processing is enhanced by emotion (Vuilleumier, 2005). Connections from the amygdala are thought to be implicated in the processing of facial emotions (Garvert et al., 2014), allowing rapid “feedback” signals from subcortical areas to the visual cortex (Pourtois et al., 2013). In order to investigate early emotional processing, event-related potentials (ERPs) enable extremely accurate time-domain resolution. Specific activity to emotions has been described in a wide range of EEG studies, showing that emotions are processed automatically and rapidly (Anderson et al., 2003) as they modulate early visual components like the P1 and the N170 (Batty and Taylor, 2003; Brenner et al., 2014; Hinojosa et al., 2015).

Visual mismatch negativity (vMMN) has been widely studied to uncover neurophysiological mechanisms involved in automatic change-detection (Kimura et al., 2011; Czigler, 2014). The vMMN is an ERP representing the pre-attentive neural mechanism involved in the processing of unexpected information (Czigler and Pató, 2009; Berti, 2011; Flynn et al., 2016). It is defined as the activity resulting from the subtraction of a standard stimulation from a deviant stimulation, usually presented in oddball paradigms. The vMMN has been recorded over posterior regions in an early latency range peaking around 100–200 ms post stimulus but also in a late latency range, between 200 and 500 ms depending on stimulation and clinical condition (see Kremlácek et al., 2016), with neural activity also reported in frontal regions (Cléry et al., 2013; Hedge et al., 2015). The vMMN has been identified in several low-level processing contexts including color, orientation, size, position, motion, luminance, and spatial frequency (Astikainen et al., 2008; Czigler and Pató, 2009; Clery et al., 2012; Cleary et al., 2013; Qian et al., 2014), but also in higher level perception (Thierry et al., 2009; Fujimura and Okanoya, 2013; Sulykos et al., 2015). Recent studies have proposed the prediction-error account as an explanation of the vMMN mechanism. According to this model, vMMN is elicited when an input does not match the prediction induced by regular and probabilistic representation. This interpretation would explain not only deviancy effects in oddball paradigms, but also violations of regularity in sequential patterns (see Kimura et al., 2011; Kimura, 2012; Vogel et al., 2015).

A decrease in ERPs amplitude to standard stimuli, called the refractoriness effect, might be observed as a consequence of stimulus repetition, either due to an adaptation mechanism, or a loss of responsiveness of activated neurons (see O'Shea, 2015; Stefanics et al., 2016). Recently, methodological considerations have addressed the question of refractoriness in oddball paradigms (Kimura et al., 2009; Stefanics et al., 2014a,b). To control for the neural habituation induced by the repeated stimulation of one specific stimulus and for low-level physical features, an equiprobable sequence (Schröger and Wolff, 1996; Kimura et al., 2009; Li et al., 2012) can be used in addition to the oddball paradigm. In such equiprobable sequences, all stimuli are presented with the same low probability of occurrence, corresponding to that of the deviant stimulus in the oddball sequence. In this case, a controlled MMN is measured by the subtraction of the evoked response to the stimulus presented as a deviant in the oddball sequence to itself in the equiprobable sequence (Kimura et al., 2009). Using an equiprobable sequence with bars of different orientations as stimuli, Kimura et al. (2009) showed that while deviant stimulus from an oddball sequence elicited both early occipital (100–150 ms) and late temporal (200–250 ms) negativities, the controlled vMMN consisted only of the late negativity. This study suggests that the early negative deflection should be considered a consequence of refractoriness and that only the late vMMN reflects memory-comparison-based change detection.

An increasing number of vMMN studies has investigated automatic change detection of socially-relevant stimuli such as gender detection (Kecskés-Kovács et al., 2013; Wang et al., 2016) or trustworthiness (Kovács-Bálint et al., 2014). Since the review of Czigler (2014), the interest for the emotion-related vMMN has resulted in a large increase in experimental studies (see Table 1).

**Table 1:** **Experimental features and results from emotional vMMN studies**.

	**Study**	**Stimuli**	**Standard**	**Deviants**	**Stimulus duration (ms)**	**Task**	**Paradigm**	***N***	**Reference**	**Amplitude measure Latency range (ms)**	**Centro-Frontal component**	**Electrodes**	**Generators**
1	Susac et al., 2004	2 identities Upright & Inverted blocks	Neutral Happy	Neutral (identity)	150	Visual *Counting target (face glasses)*	Oddball *Reverse*	8	Nose	Peak + Mean 250–300	No	PO8	
2	Zhao and Li, 2006	1 Identity	Neutral	Sad Happy	100	Auditory *Discrimination*	Oddball	14	Nose	Peak + Mean 125–365	No	P7–P8, PO7, PO8 CB1, CB2	
3	Astikainen and Hietanen, 2009	2 identities	Neutral	Fearful Happy	200	Auditory *Counting target*	Oddball	12	Nose	Peak + Mean 150–180 280–320	Yes	Fz, Cz, Pz O1, Oz, O2	
4	Susac et al., 2010	Several identities	Neutral Happy	Neutral (identity) Neutral	150	Visual *Counting target (face glasses)*	Oddball *Reverse*	5	Nose	Peak + Mean 225–350	No	PO8, FCz, Pz	
5	Gayle et al., 2012	Several identities 1 identity per block	Neutral	Sad Happy Neutral	150	Auditory *Counting target*	Oddball	45	Nose	Mean 150–425	No	PO3, PO4 PO7, PO8	
6	Kimura et al., 2012	Several identities Upright & Inverted blocks	Happy Fearful	Fearful Happy	250	Visual *Detection (face glasses)*	Oddball-like	12	Nose	Mean 275–380	No	PO8 (fearful) POZ (happy)	Frontal, temporal, limbic, occipital for late vMMN
7	Li et al., 2012	Several identities	Neutral	Sad	200	Auditory + Visual *Detection (White circle)*	Oddball Equiprobable	12	Nose	Mean 110–410	No	O1-O2 PO7, PO8 CB1-CB2	Insula, temporal, parietal and limbic
8	Stefanics et al., 2012	Several identities 4 periphically	Fearful Happy	Fearful Happy	200	Visual *Detection (Line lenght)*	Oddball	24	Average	Mean 70–390	Yes	ROI (occipital, temporal, frontal, central)	
9	Astikainen et al., 2013	Several identities	Neutral	Fearful Happy	200	Auditory *Passive listening*	Oddball Equiprobable	10 × 2	Average	ICA components 100–200	Yes	Oz, Pz, P7, P8 Fz, F3, F4	
10	Kreegipuu et al., 2013	Schematic faces Inverted for Optimal block	Neutral Angry Happy	Happy Neutral Angry	250	Visual *Detection (*letter T*)*	Oddball Optimal	11	Earlobes	Mean 100–500	Yes	ROI (occipital, temporal, frontal, central)	
11	Vogel et al., 2015	Several identities	Neutral sequence	Neutral Fearful	150	Auditory + Visual *Detection (star)*	Oddball-like	20	Average	Peak 200–400	No	P7, P8, PO7, PO8 PO9, PO10	Temporal and occipital cortex cingulate, medial temporal lobe, insula
12	Soshi et al., 2015	Schematic faces	Neutral	Happy Angry	200	Visual *Detection (White circle)*	Oddball	21	Mastoids	ICA components 55–500	Yes	O1, Oz, O2 Cz, Pz, Fz	
13	Liu et al., 2015	Several identities 2 periphically	Neutral	Happy Fearful	150	Visual *Detection (Line lenght)*	Oddball	17 high IQ 19 mid IQ	Nose	Peak 50–450	Yes	ROI (occipital, temporal, frontal)	
14	Wang et al., 2016	Several identities 4 periphically	Negative Positive	Positive Negative	200	Visual *Detection (Line lenght)*	Oddball	19	Average	Mean 80–600	Yes	ROI (occipital, temporal, frontal, central)	
15	Liu et al., 2016	Several identities 2 periphically	Neutral	Happy Fearful	150	Visual *Detection (Line lenght)*	Oddball	17 Adults 19 Adoles.	Nose	Peak 120–450	Yes	ROI (occipital, temporal, frontal)	
**CLINICAL STUDIES**													
16	Chang et al., 2010 Depression	Schematic faces Upright & Inverted Red and green	Neutral	Sad Happy	150	Visual *Counting target (green line faces)*	Oddball	15 controls 15 patients	Nose	Mean 120–200 220–320	No	P7, P8, TP7, TP8 O1, O2, M1, M2	
		6 schematic faces Red and green						10 controls 10 patients		Mean 120–200 220–360	No		
17	Csukly et al., 2013 Schizophrenia	Several identities 4 periphically	Neutral	Happy Fearful	200	Visual *Detection (Line lenght)*	Oddball	28 controls 28 patients	Average	Mean 170–390	Yes	ROI (occipital, temporal, frontal, central)	Frontal (controls)
18	Tang et al., 2013 Panic disorder	Schematic faces Red and green	Neutral	Negative Positive	150	Visual *Counting target (green line faces)*	Oddball	17 controls 12 patients	Nose	Mean 120–200 220–330	No	P7, P8, TP7, TP8 O1, O2, M1, M2	

Paradigms varied in several ways, by using different distractive tasks (Stefanics et al., 2014b for discussion), by presenting photographic pictures or schematic faces (Kreegipuu et al., 2013; Soshi et al., 2015) with one/few face identities or several face identities, but also by investigating one or two emotions as deviant stimuli within the same experiment (i.e., happy, sad, fearful and angry faces). The emotional vMMN has been reported over posterior regions, in a wide time window (100–520 ms), reflecting either successive independent early and late processing (Astikainen and Hietanen, 2009; Chang et al., 2010; Stefanics et al., 2012) or a unique pre-attentional emotional change detection phenomenon (Gayle et al., 2012; Kimura et al., 2012; Vogel et al., 2015; Wang et al., 2016). In line with right-hemisphere dominance in emotional processing previously described in ERPs (Batty and Taylor, 2003), emotional vMMN findings suggest an asymmetric response to emotional deviants with stronger responses observed in the right hemisphere (Zhao and Li, 2006; Gayle et al., 2012; Li et al., 2012; Astikainen et al., 2013). In addition to the posterior vMMN, centro-frontal activity has also been depicted (Stefanics et al., 2012; Csukly et al., 2013; Wang et al., 2016), suggesting that mechanisms of automatic visual change detection should be generalized to a broader region.

Source analyses have recently suggested that prefrontal areas are implicated in emotional deviancy processing (Kimura et al., 2012; Csukly et al., 2013), while other studies did not find prefrontal sources of the emotion-related vMMN (Li et al., 2012; Vogel et al., 2015).

To our knowledge, only two studies have compared emotional deviancy to neutral deviancy in order to disentangle the detection of emotional and neutral change (Gayle et al., 2012; Vogel et al., 2015). In Gayle et al. (2012), neutral deviants were obtained by applying a green filter to the neutral standard stimulus, changing considerably non-facial low-level features. Vogel et al. (2015) used a visual oddball-like paradigm in which stimuli were paired face photographs with a neutral or fearful expression. Results showed a latency advantage and greater amplitude for emotional deviancy compared to neutral deviancy.

The growing consideration in methodological issues has imposed particular attention in applying rigorous procedures to control for low-level features and refractoriness while recording mismatch processes. Only three studies have used an equiprobable sequence (Li et al., 2012; Astikainen et al., 2013) or an optimal paradigm (Kreegipuu et al., 2013) as control conditions for refractoriness, using face photographs or schematic faces. Li et al. (2012) showed that oddball designs elicited an earlier and larger vMMN compared to the equiprobable paradigm in the 110–210 ms latency range. Similarly, Astikainen et al. (2013) performed an independent component analysis (ICA) and revealed two components at 130 and 170 ms in both the oddball and the equiprobable sequences; with scalp distribution differences across sequences for the 130 ms component only. It was suggested that this first deflection reflected regularity violations and that the second component at 170 ms reflected emotion processing, although this study did not include a neutral deviant for comparison. So far, no study has combined a control for emotional value with a control for refractoriness. Visual change detection and emotional face processing both rely on automatic and rapid attentional systems (Brosch et al., 2011). The present study aims at determining whether the automatic detection of changes in emotional expression involves these two distinct and independent processes or whether it is subserved by an additional specific unified process. To determine the specificity of emotional change detection as compared to visual-neutral change detection, the neural activities elicited by emotional face and neutral face deviants were compared. Moreover, to control for refractoriness, stimuli were presented in both oddball and equiprobable sequences. If emotional change detection results from the co-activation of visual change detection and emotion processing, then the neutral and emotional vMMN responses should partly overlap. Conversely a system specifically dedicated to the processing of emotional change would lead to different responses in shape and topographical distribution.

## Materials and methods

### Participants

Sixteen healthy adults (6 females) participated in this study. Two subjects were excluded because of poor behavioral performance (see Behavioral Results). The remaining 14 participants (4 females) were included in ERPs and MMNs analyses. Mean age was 24.2 years (*SD* = 4.1) and all participants had normal or corrected-to-normal vision and no psychiatric or neurological disorders. The protocol was approved by the ethical committee board of the Tours University Hospital. Participants gave informed written consent and received monetary compensation for their participation. The study was conducted according to the ethical principles of the Declaration of Helsinki.

### Stimuli and procedures

**Figure 1:**
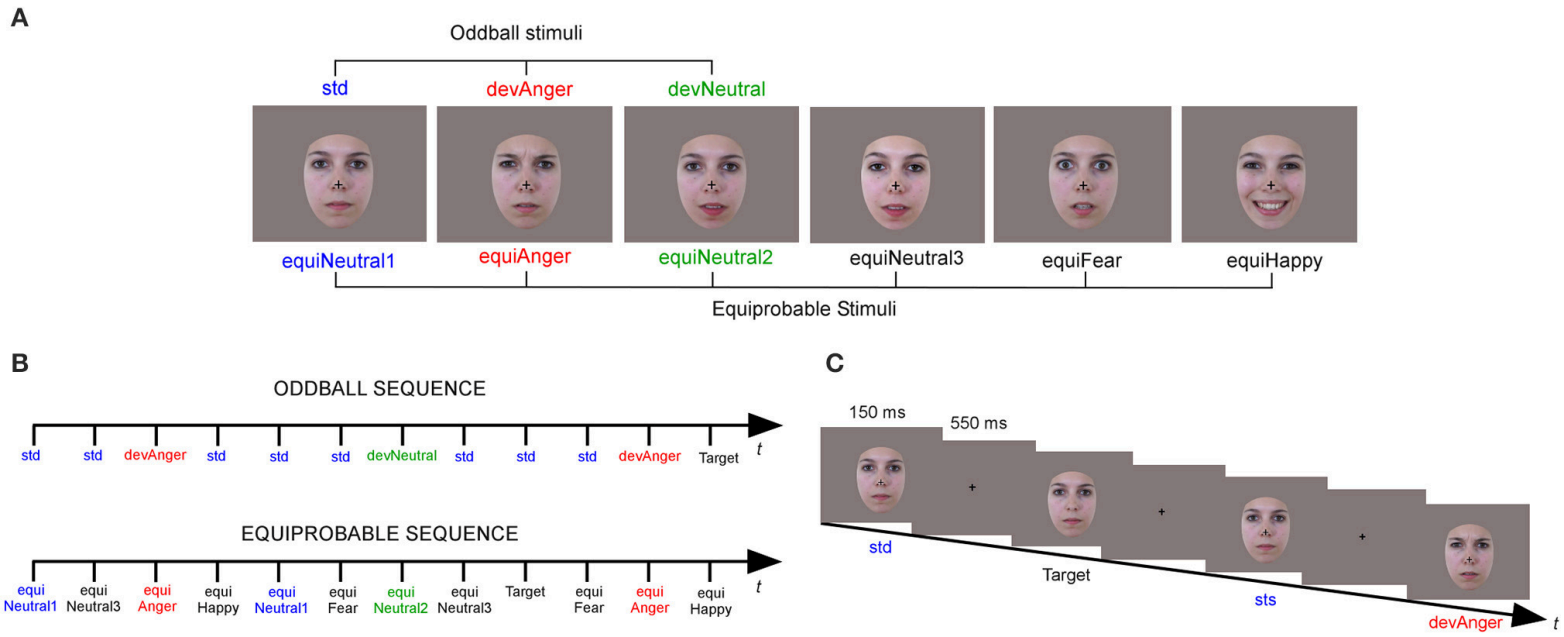
**Illustration of the six stimuli (A)** presented in the oddball sequence (Neutral standard: std; Emotional deviant: devAnger; Neutral deviant: devNeutral) and in the equiprobable sequence (Neutral 1, 2, and 3: equiNeutral1, equiNeutral2, and equiNeutral3 respectively; Emotional angry, fearful and happy: equiAnger, equiFear and equiHappy, respectively). An illustration of the oddball and equiprobable sequence **(B)**. A task schematic of the oddball sequence **(C)** shows the time course of the stimulus presented for 150 ms, followed by the cross on a gray screen displayed for 550 ms; SOA = 700 ms, and a Target stimulus (Neutral standard without cross).

Stimuli were six photographs of the same actress (Figure 1A). They were presented in two sequences: an oddball and an equiprobable sequence (Figure 1B). In the oddball sequence, a neutral expression was presented as the standard stimulus (std), with a probability of occurrence *p* = 0.80. Photographs of the same actress expressing anger or with a different neutral expression (with the mouth slightly opened) were presented as the angry deviant (devAnger, *p* = 0.10) and the neutral deviant (devNeutral, *p* = 0.10) respectively. In the equiprobable sequence, six stimuli were presented: the three stimuli of the oddball sequence and three other facial expressions. Altogether the equiprobable sequence included angry (the same stimulus as the angry deviant: equiAnger), fearful and happy faces (equiFear, equiHappy respectively), and three neutral faces (equiNeutral1: same as the standard stimulus, equiNeutral2: same as the neutral deviant and equiNeutral3) presented pseudo-randomly (*p* = 0.16 each), avoiding immediate repetition. Responses to the equiFearful, equiHappy and equiNeutral3 stimuli were not further analyzed as these stimuli were added to respect the design of the equiprobable sequence.

Stimuli were behaviorally validated for their emotional significance and arousal. A different group of 18 participants (mean age = 25.8 ± 7.1) were asked to identify and to provide the emotional intensity (1–5) of each stimulus. Stimuli of interest were presented with other emotional and neutral stimuli (total *N* = 26). Accuracy in emotion identification was 78% for anger (devAnger, equiAnger), fear (equiFear), and neutral (devNeutral, equiNeutral2), 100% for happy and neutral (std, equiNeutral1) and 89% for equiNeutral3. The mean arousal rating for all the stimuli was 2.9 (*SD* = 0.8), 2.9 for anger, 4.2 for happy, 1.85 for fear, 3 for neutral (std, equiNeutral1), 2.7 for neutral (devNeutral, equiNeutral2) and 2.4 for equiNeutral3.

Participants sat comfortably in an armchair with stimuli projected on a screen located 120 cm in front of them. Stimuli were presented using Presentation® software in the central visual field (visual angle: width = 5.7°, height = 8.1°) for 150 ms with a 550 ms inter-stimulus interval (Figure 1C). The oddball sequence comprised 1575 stimuli and the equiprobable sequence 924 stimuli. Total recording time lasted 30 min. As in previous vMMN studies, in order to study automatic change detection, subjects were asked to focus on a concurrent visual task. Target stimuli consisted of face stimuli in which a black fixation cross on the nose, otherwise present, disappeared. Participants were instructed to look at the fixation cross and to press a button as quickly as possible when the cross disappeared. Targets occurred on neutral standards in the oddball sequence, and on any stimulus in the equiprobable sequence (*p* = 0.05). All subjects were monitored with a camera during the recording session to ensure compliancy to the task.

### EEG recording

EEG data were recorded using a 64-channel ActiveTwo system (BioSemi®, The Netherlands). To record the electro-oculographic activity, two electrodes were applied on the left and right outer canthi of the eyes and one below the left eye. An additional electrode was placed on the tip of the nose for offline referencing. During recording, impedances were kept below 10 kΩ. EEG signal was recorded with a sampling rate of 512 Hz and filtered at 0–104 Hz.

### Behavioral data analysis

Accuracy and false alarms (FAs) in the target detection task (concurrent task) were analyzed and the sensitivity index, *d'* = z-score (% correct responses) − z-score (FAs) was measured to evaluate the degree of attention of the participants during the task.

### EEG data analysis

**Figure 2:**
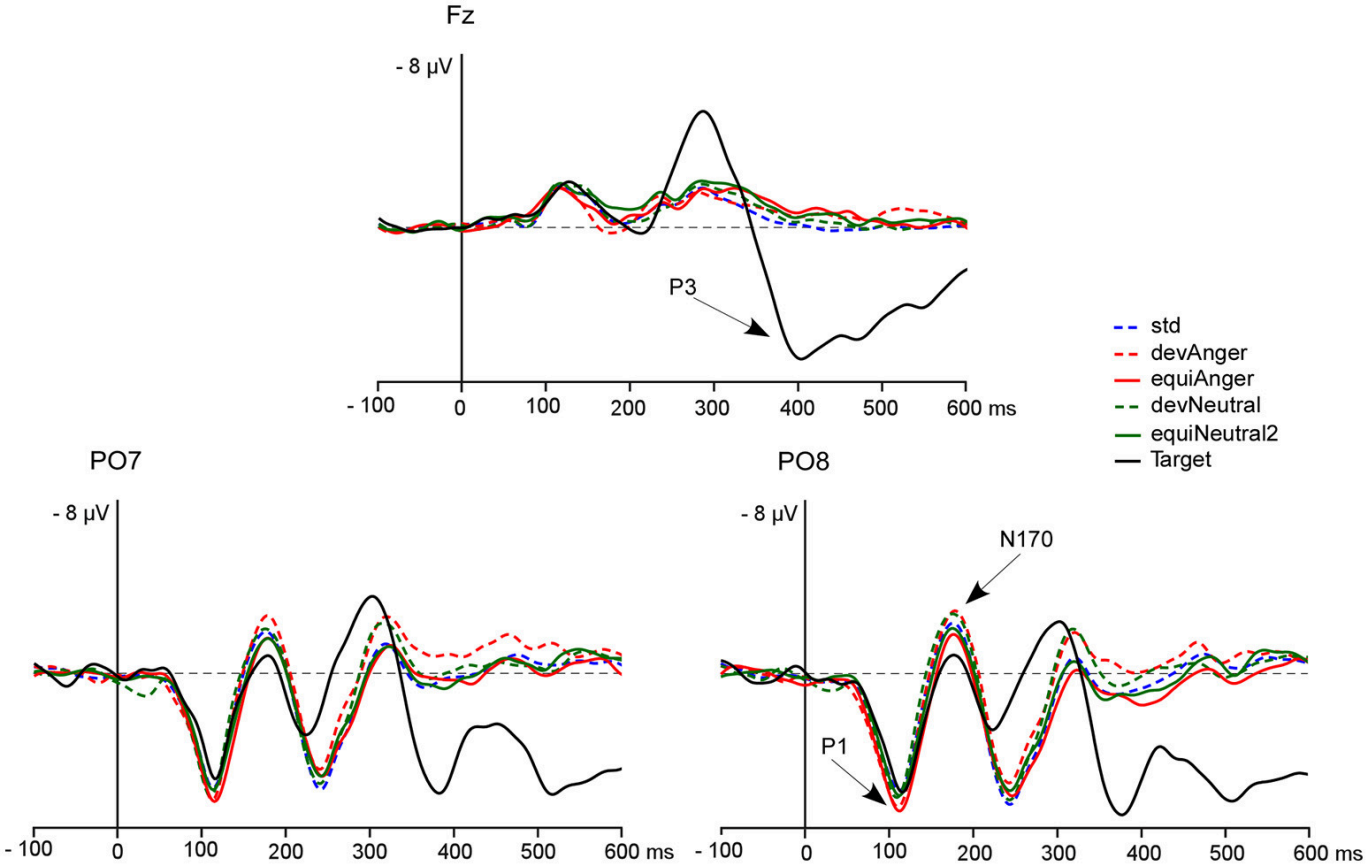
**The grand-average ERPs at Fz, PO7, and PO8 elicited by targets (black line), std (blue dotted line), devAnger (red dotted line), equiAnger (red line), devNeutral (green dotted line), and equiNeutral2 (green line)**. P1, N170, and P3 are indicated by arrows on the PO8 and Fz electrodes. Over the PO7 electrode P1 peak presented a mean amplitude of 6.6 μV (*SD* = 2.6) for devAnger, 6.7 ± 3.0 μV for equiAnger, 6.6 ± 2.9 μV for devNeutral and 6.5 ± 2.2 μV for equiNeutral2. For N170 mean amplitude was −3.5 ± 4.8 μV for devAnger, −2.4 ± 4.2 μV for equiAnger, −3.2 ± 4.2 μV for devNeutral and −2.3 ± 4.8 μV for equiNeutral2.

A 0.3 Hz digital high-pass filter was applied to the EEG signal. Ocular artifacts were removed by applying ICA as implemented in EEGLab. Blink artifacts were captured into components that were selectively removed via the inverse ICA transformation. Thirty-two components were examined for artifacts and one or two components were removed in each subject. Muscular and other recording artifacts were discarded manually. EEG data were recorded continuously and time-locked to each trial onset. Trials were extracted over a 700 ms analysis period, from 100 ms pre-stimulus to 600 ms post-stimulus. ERPs were baseline corrected and digitally filtered with a low-pass frequency cutoff of 30 Hz. The first three trials of a sequence, as well as trials occurring after deviant or target stimuli were not included in averaging. Each ERP was computed by averaging all trials of each stimulus type (see Figure 2) from the oddball sequence (std, devAnger, devNeutral) and from the equiprobable sequence (equiAnger, equiNeutral2). For each stimulus of interest the average of artifact-free trials was: 672 ± 95 (std), 127 ± 16 (devAnger), 127 ± 15 (devNeutral), 117 ± 22 (equiAnger), and 119 ± 23 (equiNeutral2). Responses were then analyzed and compared with the ELAN software (Aguera et al., 2011). In the oddball sequence brain activity specifically elicited by emotional or neutral automatic change detection were obtained by subtracting ERPs to std stimuli from ERPs to devAnger or devNeutral stimuli obtaining respectively an emotional vMMN (anger vMMN) and a neutral vMMN. Likewise, controlled vMMNs were calculated as ERPs to devAnger and ERPs to devNeutral stimuli minus the responses elicited by the same emotional (equiAnger) and neutral (equiNeutral2) stimuli respectively presented in the equiprobable sequence (anger-control vMMN = devAnger − equiAnger; neutral-control vMMN = devNeutral − equiNeutral2).

Group grand average difference waveforms across participants were examined in order to establish deflections of interest. Specific brain activity related to emotion deviants, compared to neutral deviants as well as the effect of control were investigated, by comparing oddball emotional and neutral vMMN to emotional and neutral controlled vMMN. For posterior vMMN measurement, 10 electrodes were selected (O1, O2, PO3, PO4, PO7, PO8, P7, and P8) and for central activity, nine electrodes were selected (CP1, CPz, CP2, C1, Cz, C2, FC1, FCz, and FC2) based on previous studies and visual inspection.

### Statistical ERPs analyses

P1 and N170 components were measured on the ERPs evoked by each stimulus type (devAnger, devNeutral, equiAnger, equiNeutral2). Peak amplitudes were measured in the 80–160 ms latency range at O1, Oz, and O2 for P1, and in the 130–210 ms latency range at PO7 and PO8 for N170. Data were analyzed with repeated-measures analysis of variance (ANOVA) with Emotion (anger vs. neutral) x Sequence (oddball vs. controlled) × Site as within-subject factors. For significant results, the effect sizes are shown as $\eta_{p}^{2}$.

### Statistical MMNs analyses

First, for each condition (i.e., anger and neutral deviants), both oddball and controlled MMN responses were tested for significance by comparing ERP amplitudes to 0, using Student *t*-test corrected for multiple comparisons (Guthrie and Buchwald, 1991) at each electrode and each time point. This analysis provides information about the presence of meaningful deflections. Subsequently, mean amplitude was analyzed in selected time-windows using ANOVA (described below). When necessary, ANOVAs results were corrected with the Greenhouse-Geisser procedure and *post-hoc* analyses were corrected with a Bonferroni correction. For significant results, the effect sizes are shown as $\eta_{p}^{2}$. When interactions between conditions and electrodes were found, topographic differences were specifically tested on amplitude-normalized data (McCarthy and Wood, 1985). Measurements for each subject were normalized by subtracting the minimum value from each electrode value, and by dividing it by the difference between maximum and minimum.

#### Control effect

Over posterior sites, mean amplitude was measured in the 100–200 ms latency range. A repeated-measure ANOVA was performed including Emotion × Sequence × Sites (occipital: O1, O2; medial parieto-occipital: PO3, PO4 and lateral parieto-occipital: PO7, PO8) × Hemispheres (left: O1, PO3, PO7; right: O2, PO4, PO8).

**Figure 3:**
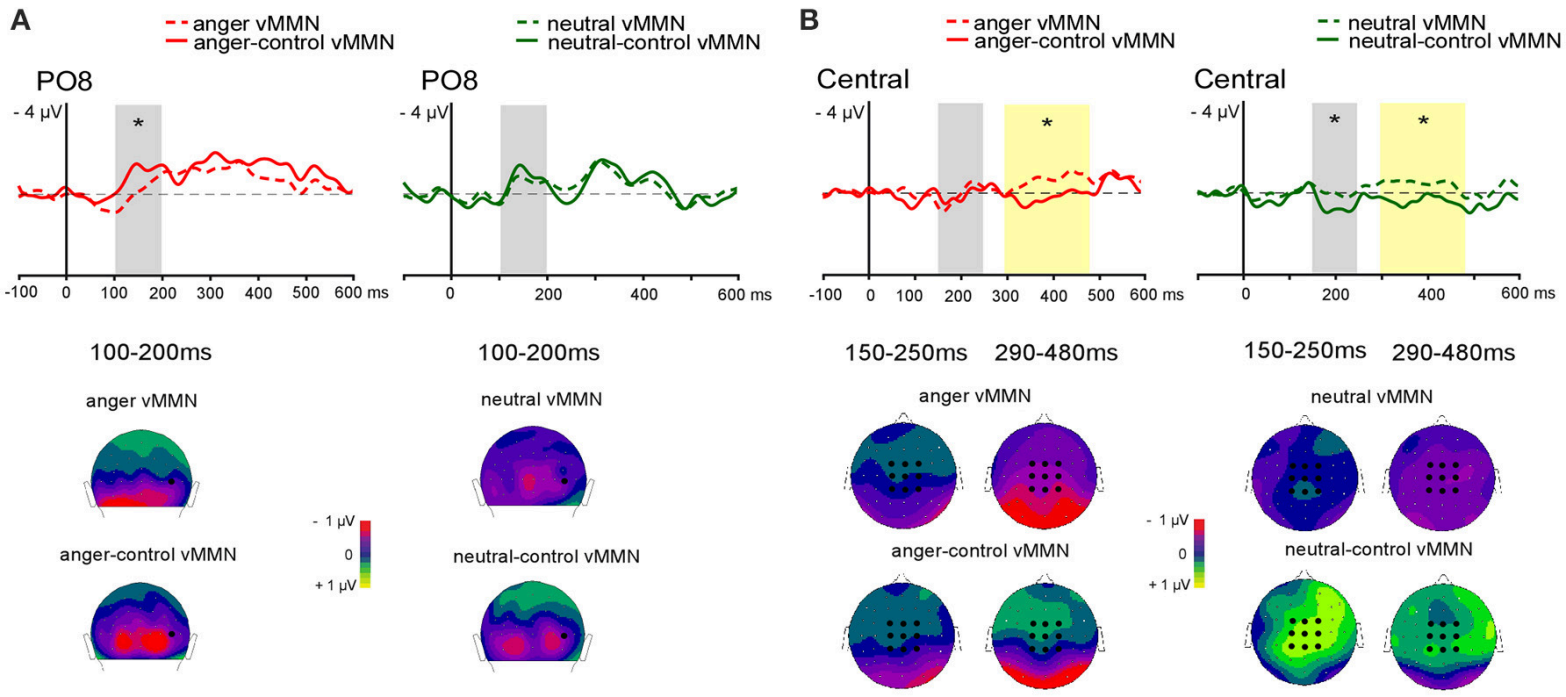
**Grand-average vMMN comparaison between oddball and controlled vMMN at posterior site at PO8 (A)** and averaged central electrodes **(B)**. Emotional vMMN on the left and neutral vMMN on the right. Dashed line represents oddball vMMN and continued line represents controlled vMMN. Analyses were performed in two time windows for early (gray panel) and late (yellow panel) effect. Below, 2D scalp distributions (back view for posterior activity with black dot showing PO8 position; top view for central activity with black dots showing averaged electrodes). Time windows are displayed for posterio activity (100–200 ms), and for central activity (150–250 and 290–480 ms). Significant differences are indicated by asterisks: ^*^p < 0.05.

For the central vMMN the averaged value of central electrodes (see Figure 3B) was analyzed in the 150–250 ms and in the 290–480 ms latency ranges. A repeated-measure ANOVA was performed with Emotion (anger vs. neutral) and Sequence (oddball vs. controlled) as factors.

#### Emotional effect

**Figure 4:**
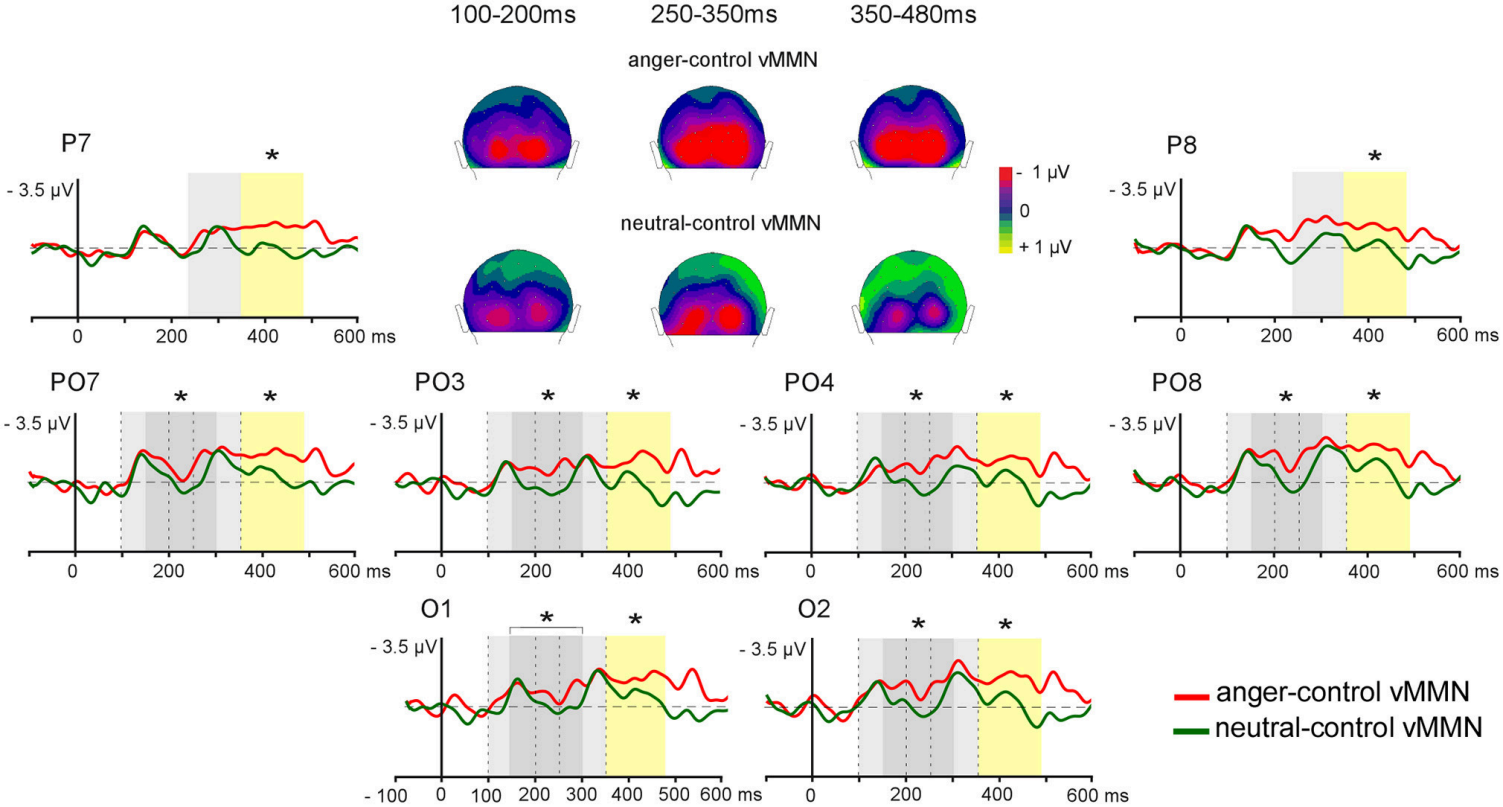
**Controlled grand-average vMMNs (anger-control vMMN, neutral-control vMMN) elicited by emotional deviant (red line) and by neutral deviant (green line) over the parieto-occipital region, with time windows of significant early difference (150–300 ms, dark gray panel) and late difference (350–480 ms, yellow panel), and commun activity (light gray pannels for 100–200, 250–350 ms time windows)**. 2D scalp distributions (back view) of averaged time windows for common activity (100–200, 250–350 ms) and sustained emotional effect (350–480 ms). Significant differences are indicated by asterisks: ^*^p < 0.05.

Specific response to emotional change was investigated by comparing mean amplitude of controlled vMMNs (anger-control vMMN vs. neutral-control MMN) in several latency ranges based on visual inspection (see Figure 4). For early vMMNs, mean amplitudes were measured in the 100–200 ms latency range corresponding to the common neutral and emotional response peaks. In order to measure the differential activity and investigate specific response to emotional deviants a following latency range was selected (150–300 ms). Within these time-windows ANOVAs were performed including Emotion (angry; neutral) × Sites (occipital: O1, O2; medial parieto-occipital: PO3, PO4, and lateral parieto-occipital: PO7, PO8) × Hemispheres (left: O1, PO3, PO7; right: O2, PO4, PO8).

Two other time-windows were selected in order to analyze late vMMN corresponding to activity common to both conditions (250–350 ms) and activity specific to emotional deviants (350–480 ms). The same statistical analyses were performed on mean amplitude over each time window measured over four Sites (occipital: O1, O2; medial parieto-occipital: PO3, PO4; lateral parieto-occipital: PO7, PO8; parietal: P7, P8) and in the two Hemispheres (left: O1, PO3, PO7, P7; right: O2, PO4, PO8, P8).

## Results

### Behavioral results

Because of poor detection rate and false alarms, two subjects were not included in the EEG analysis (*d'* = 0.75 and 1.29, respectively). For the remaining 14 subjects, the distractive task was performed correctly (*d'* = 4.72; *SD* = 0.77) with a mean accuracy rate of 90.5; *SD* = 10% (95% confidence interval 85.3–95.8).

### ERPs results

#### P1

A significant effect of Sequence was found regarding P1 amplitude [*F*_(1, 13)_ = 5.16, *p* = 0.04, $\eta_{p}^{2}$ = 0.28] as it was smaller in response to deviants in the oddball sequence (devAnger, devNeutral) compared to the same stimuli presented in the equiprobable sequence (equiAnger, equiNeutral2). The effect of Emotion was significant in both sequences [*F*_(1, 13)_ = 11.76, *p* < 0.01, $\eta_{p}^{2}$ = 0.47] with greater amplitude for emotional stimuli compared to neutral stimuli (see Figure 2). There was no interaction.

#### N170

The effect of Sequence was significant [*F*_(1, 13)_ = 12.27, *p* < 0.01, $\eta_{p}^{2}$ = 0.48] as deviants presented in the oddball sequence elicited a larger response, compared to the same stimuli presented in the equiprobable sequence. There were neither an effect of Emotion, nor interaction.

### MMNs results

Student *t*-tests for each vMMN condition at each time point and electrode revealed deflections significantly different from 0 (*p* < 0.05) between 100 and 500 ms.

#### Control effect

##### Posterior vMMN

In the early 100–200 ms latency range a significant interaction between Emotion and Sequence was found [*F*_(1, 13)_ = 6.74, *p* = 0.02, $\eta_{p}^{2}$ = 0.34] with controlled vMMN being larger than oddball vMMN for the emotional deviant only (*p* = 0.01). A main effect of Site was found [*F*_(2, 26)_ = 5.34, *p* = 0.01, $\eta_{p}^{2}$ = 0.29, see Figure 3A]. However, the interaction between Sequence and Site was significant [*F*_(2, 26)_ = 4.40, *p* = 0.02, $\eta_{p}^{2}$ = 0.25]. *Post-hoc* analysis revealed a larger amplitude over lateral parieto-occipital electrodes compared to medial parieto-occipital electrodes for controlled vMMN only (*p* < 0.001) as well as a greater amplitude for controlled vMMN compared to oddball vMMN over the lateral parieto-occipital electrodes (*p* < 0.001).

##### Central vMMN

In the 150–250 ms latency range, a significant interaction between Emotion and Sequence was found [*F*_(1, 13)_ = 6.80, *p* = 0.02, $\eta_{p}^{2}$ = 0.34]. *Post-hoc* analyses revealed larger amplitude for neural-control vMMN compared to neutral vMMN (*p* = 0.014) and anger-control vMMN (*p* = 0.009).

In the 290–480 ms latency range, analysis revealed a significant main effect of Sequence [*F*_(1, 13)_ = 18.37, *p* < 0.001, $\eta_{p}^{2}$ = 0.59], with oddball vMMN being larger than controlled vMMN.

#### Emotional effect

In the posterior region, early (~140 ms) and late (~310 ms) peaks were identified for both anger-control vMMN and neutral-control vMMN. In order to fully characterize differences between anger-control vMMN and neutral-control vMMN, mean amplitude over time was measured around the two peaks (100–200 ms; 250–350 ms) and in two additional time windows (150–300 ms; 350–480 ms) over posterior regions (Figure 4).

In the early latency range (100–200 ms) the effect of Emotion was not significant [*F*_(1, 13)_ < 1, *p* = 0.34], highlighting a similar processing of both deviants. The effect of Site was significant [*F*_(2, 26)_ = 7.20, *p* = 0.04, $\eta_{p}^{2}$ = 0.36], with amplitude over lateral parieto-occipital sites being greater than over medial parieto-occipital sites (*p* = 0.03).

In the 150–300 ms time-window a significant difference between anger-control vMMN and neutral-control vMMN was found [*F*_(1, 13)_ = 5.10, *p* = 0.04, $\eta_{p}^{2}$ = 0.28] with anger-control vMMN being larger. The effect of Site was significant [*F*_(2, 26)_ = 4.11, *p* = 0.03, $\eta_{p}^{2}$ = 0.24], with amplitude over lateral parieto-occipital sites being larger than over medial parieto-occipital sites (*p* = 0.02).

In the 250–350 ms latency range, the main effect of the Emotion was not significant [*F*_(1, 13)_ = 3.20, *p* = 0.10], revealing similar anger-control vMMN and neutral-control vMMN responses. Here, ANOVA revealed a significant effect of Site [*F*_(3, 39)_ = 4.84, *p* < 0.01, $\eta_{p}^{2}$ = 0.27], with amplitude over lateral parieto-occipital sites being larger than over parietal sites (*p* = 0.03).

In the 350–480 ms time-window, the effect of Emotion was significant [*F*_(1, 13)_ = 5.40, *p* = 0.04, $\eta_{p}^{2}$ = 0.29] as anger-control vMMN was greater than neutral-control vMMN. The effect of Site was also significant [*F*_(3, 39)_ = 3.82, *p* = 0.02, $\eta_{p}^{2}$ = 0.23]. *Post-hoc* analyses did not reveal significant effects, however amplitude tended to be larger at lateral parieto-occipital than at medial parieto-occipital and parietal sites (*p* = 0.06, both). Figure 4 represents the electrodes of interest and the time windows revealing the early difference between Emotion conditions (gray panel) and the late difference (yellow panel) with greater amplitude for anger-control vMMN than neutral-control vMMN. Scalp distributions show the similar activity between the neutral and the emotional deviants around the first and second peaks (100–200, 250–350 ms, respectively) and the sustained negative activity for angry deviants only in the time windows following the peaks (150–300, 350–480 ms).

## Discussion

In the present study we investigated pre-attentive processing of emotional change via a controlled vMMN paradigm. The main aim was to disentangle automatic detection of emotional and neutral changes by comparing vMMN elicited by an emotional expression deviant to vMMN elicited by a neutral deviant. Controlled vMMN was measured by presenting an oddball sequence and a control equiprobable sequence to participants (Schröger and Wolff, 1996; Kimura et al., 2009; Li et al., 2012). This allowed controlling for refractoriness due to standard stimulus repetition in the oddball sequence and to remove neural response to low-level differences. To the best of our knowledge, this is the first study disentangling emotional and neutral expression change detection within a highly controlled paradigm.

### Control for refractoriness

In order to investigate how refractoriness impacts emotional and neutral change detection, oddball, and controlled vMMNs were compared. Over posterior regions (100–200 ms latency window) controlled vMMN mean amplitude was larger than oddball vMMN for emotional deviants. Differences between oddball and controlled vMMNs to a sad deviant expression were previously reported in the 110–210 ms latency window (Li et al., 2012).

In a similar time window, Astikainen et al. (2013) found that both oddball and controlled emotional vMMNs elicited two early components at 130 ms and at 170 ms. As different scalp distributions between sequences were found for the 130 ms component only, authors suggested that this first component reflected regularity violations while the 170 ms component reflected emotional processing. Our data seem in accordance with this interpretation as the anger-control vMMN (see Figure 3A) displayed a biphasic shape in the 100–200 ms time-window that might correspond to the two distinct components described by Astikainen et al. (2013), with the first deflection being influenced by the effect of the control.

Additionally, differences between the oddball vMMN and the controlled vMMN were observed centrally, suggesting the involvement of pre-attentional mechanisms (Astikainen and Hietanen, 2009; Stefanics et al., 2012; Csukly et al., 2013) that appears to be influenced by the refractoriness effect.

### Sustained effect specific to emotional deviants

We provide additional information on automatic emotional processing by disentangling the specificity of emotional change detection from neutral change detection. The present study shows that both neutral and emotional deviants elicited early and late vMMN, peaking at ~140 and ~310 ms respectively. Early and late vMMNs have previously been interpreted as two different stages reflecting either modulation of N170 and P250 (Chang et al., 2010) or detection and pre-attentional processing (Astikainen and Hietanen, 2009). However, an emotional vMMN peak interpreted as a unique detection processing has often been reported around the 260–350 ms latency range over occipital or parieto-occipital electrodes (Susac et al., 2004, 2010; Gayle et al., 2012; Kimura et al., 2012; Vogel et al., 2015) and sometimes even earlier (Li et al., 2012; Stefanics et al., 2012; Liu et al., 2015). This might depend on the design choices but also on different investigation strategies as the two steps are less recognizable in the emotional vMMN than in the neutral vMMN as it is more sustained. In our study the neutral vMMN enabled the identification of two peaks, in support of the hypothesis of a two-step vMMN response (Zhao and Li, 2006; Astikainen and Hietanen, 2009; Stefanics et al., 2012; Flynn et al., 2016).

The early emotional vMMN has previously been suspected reflecting modulation of N170 (Chang et al., 2010). However, in the present study, an effect of Emotion was found on the P1 component only with peak amplitude being larger to emotional stimuli than to neutral stimuli (see Batty and Taylor, 2003; Pourtois et al., 2004; Vlamings et al., 2009; Liu et al., 2016) in both oddball and equiprobable sequence. The present data are consistent with previous findings where negative facial expression modulated the P1 (Batty and Taylor, 2003; Pourtois et al., 2004; Brosch et al., 2008) but not the N170 component (Batty and Taylor, 2003). Thus, P1 component and not only the face-specific N170 should be considered when investigating the influence of emotional processing on face related vMMN. These findings also suggest that the early processing of the emotional content of faces is not simultaneously converted into emotional change detection and that the emotional mismatch process occurs only at a later stage.

Differences in vMMN responses might also be related to the emotion selected as deviant. Although both positive and negative emotions were found to elicit vMMN, happy faces are chosen more often than other emotions (Zhao and Li, 2006; Astikainen and Hietanen, 2009; Gayle et al., 2012; Li et al., 2012; Astikainen et al., 2013; Kreegipuu et al., 2013; Soshi et al., 2015). Depending on the valence of the emotional deviant, the emotional vMMN was associated to different peak latencies and scalp distributions (Kimura et al., 2012; Astikainen et al., 2013). Supported by previous ERPs findings (Batty and Taylor, 2003), a latency advantage has been reported when happy deviants were compared to negative deviants (Zhao and Li, 2006; Astikainen and Hietanen, 2009). In contrast, a shorter vMMN latency for fearful faces compared to happy faces was also identified (Kimura et al., 2012; Stefanics et al., 2012; Wang et al., 2016).

In the present study, we provide evidence for an enhanced response to anger compared to neutral deviant in line with a negative-bias showing a greater vMMN amplitude for automatic detection of sadness (Zhao and Li, 2006; Gayle et al., 2012), fear (Stefanics et al., 2012), and anger (Kuldkepp et al., 2013, with schematic faces). Importantly, other studies did not report a significant difference in the vMMN amplitude to positive or negative emotions (Astikainen and Hietanen, 2009; Astikainen et al., 2013), suggesting that differences in protocols (i.e., concurrent task, paradigm, stimuli) modulate the face-related vMMN.

Two studies have provided the comparison between neutral and emotional deviants (Gayle et al., 2012; Vogel et al., 2015). However, in Gayle et al. (2012) although the sad face deviant elicited greater vMMN response compared to happy and neutral deviants, the neutral deviant was computed by applying a green filter to the standard stimulus, therefore changing low-level stimulus features in addition to facial expression *per se*. Vogel et al. (2015) used a visual oddball-like paradigm where the standard stimulation consisted of a sequence with two neutral faces. A change in the emotional expression (fearful expression) or in the identity of the second stimulus was used as the emotional change or sequential change respectively. In this way, Vogel et al. (2015) showed a latency advantage and a greater amplitude for the emotional deviant, compared to the neutral sequence deviancy.

Previous studies (Gayle et al., 2012; Li et al., 2012; Vogel et al., 2015) reported a right-hemisphere lateralization of the emotional vMMN and of its neural generators (Kimura et al., 2012) to sad, happy and fearful deviants. In the present study the vMMN responses were instead symmetric and no right-hemisphere dominance was found. This is in line with another vMMN study using angry faces as deviants (Kreegipuu et al., 2013), as well as with ERP findings indicating that this lateralization is not consistently reported in the N170 latency range (Batty and Taylor, 2003; Hinojosa et al., 2015). This lateralization of the emotional responses might thus depend on the paradigm used and on the specific valence of the stimuli (see for discussion, Stefanics et al., 2012).

In our study, angry deviants were not relevant to the target detection task and were automatically detected as fast as neutral deviants. Thus, a common change detection biphasic response was observed in the occipital and parieto-occipital region in the 150–300 and 350–480 ms latency ranges consistently with previous evidence on face-related vMMN. Importantly, a sustained response was observed for emotional deviants represented by greater mean vMMN amplitude following the early and the late vMMN peaks. These results suggest that this two-step vMMN response is partly devoted to automatic change detection and reorienting attention regardless of the emotional valence of the stimulus. A rapid detection of a visual change would thus be necessary prior to an emotion-specific processing, at least regarding the emotion (anger) used in the present study. Findings suggests that the detection of emotional change relies on both a broad visual change detection process and an emotion-specific process represented by the sustained activity.

## Conclusion

The present study allows to identify a two-step vMMN and to disentangle what is proper to automatic emotion detection, thus contributing to the understanding of vMMN processing and of automatic attentional mechanisms involved in emotional processing. Thus, automatic detection of changes in emotional expression would involve the activation of two distinct pre attentional systems, the visual change detection mechanism and an additional emotional processing. This sustained emotional vMMN response is consistent with previous studies (Holmes et al., 2009, 2014) proposing that angry faces hold attention even when they are not directly relevant to an ongoing task. From an evolutionary perspective, avoiding danger by orienting the attention to salient stimuli and by detecting changes in the environment is a fundamental requisite for survival. Consistent with emotions capturing attention (see Carretié, 2014), we show that while emotional and neutral changes are both automatically detected, detection of angry expressions involve enhanced processing. Clinical application (see Kremlácek et al., 2016) of the vMMN would benefit in future studies from investigations on the emotional vMMN, especially in psychiatric conditions defined by both attentional and emotional impairments (i.e., autism).

## Author contributions

KK performed the acquisition, analysis, interpretation of the data and wrote the first draft of the manuscript; JC and HC participated in the acquisition and design of the protocole; EH and FB participated in the inclusion of the participants; SR made a substantial contribution to the programming of the stimulus sequence, to the acquisition and analysis of data; ML and MB participated in the analysis, interpretation of the data and writing of the manuscript; MG designed the protocol, guided the analysis, contributed to the interpretation of the data and the writing of the manuscript. All authors participated in revising the manuscript critically for important intellectual content.

## Funding

This study was supported by the French Agence Nationale de la Recherche (ANR- 12-JCJC-SH2-0001-01 AUTATTEN) and a grant of the “Région Centre” (KK).

### Conflict of interest statement

The authors declare that the research was conducted in the absence of any commercial or financial relationships that could be construed as a potential conflict of interest.
